# Nephronectin plays critical roles in Sox2 expression and proliferation in dental epithelial stem cells via EGF-like repeat domains

**DOI:** 10.1038/srep45181

**Published:** 2017-03-27

**Authors:** Chieko Arai, Keigo Yoshizaki, Kanako Miyazaki, Kan Saito, Aya Yamada, Xue Han, Keita Funada, Emiko Fukumoto, Naoto Haruyama, Tsutomu Iwamoto, Ichiro Takahashi, Satoshi Fukumoto

**Affiliations:** 1 Section of Orthodontics and Dentofacial Orthopedics, Division of Oral Health, Growth and Development, Faculty of Dental Science, Kyushu University, Fukuoka 812-8582, Japan; 2 Division of Pediatric Dentistry, Department of Oral Health and Development Sciences, Tohoku University Graduate School of Dentistry, Sendai 980-8575, Japan; 3 Department of Pediatric Dentistry, Institute of Biomedical Science, Tokushima University Graduate School, Tokushima 770-8540, Japan

## Abstract

Tooth development is initiated by epithelial-mesenchymal interactions via basement membrane (BM) and growth factors. In the present study, we found that nephronectin (Npnt), a component of the BM, is highly expressed in the developing tooth. Npnt localizes in the BM on the buccal side of the tooth germ and shows an expression pattern opposite that of the dental epithelial stem cell marker Sox2. To identify the roles of Npnt during tooth development, we performed knockdown and overexpression experiments using *ex vivo* organ and dental epithelial cell cultures. Our findings showed that loss of Npnt induced ectopic Sox2-positive cells and reduced tooth germ size. Over expression of Npnt showed increased proliferation, whereas the number of Sox2-positive cells was decreased in dental epithelial cells. Npnt contains 5 EGF-like repeat domains, as well as an RGD sequence and MAM domain. We found that the EGF-like repeats are critical for Sox2 expression and cell proliferation. Furthermore, Npnt activated the EGF receptor (EGFR) via the EGF-like repeat domains and induced the PI3K-Akt signaling pathway. Our results indicate that Npnt plays a critical scaffold role in dental epithelial stem cell differentiation and proliferation, and regulates Sox2 expression during tooth development.

To understand the mechanisms of tooth development, it is important to examine the roles of tissue interactions in organ morphogenesis regulated by epithelial-mesenchymal interactions, such as those occurring in hair, lung, mammary gland, and kidney tissues. Tooth development is modulated by reciprocal interactions between the neural crest-derived mesenchyme and oral ectoderm^1^,^2^,^3^. In mice, tooth morphogenesis is initiated by thickening of the dental epithelium to form dental placode, followed by invagination into surrounding mesenchyme on embryonic day (E) 11.5. Continuation of this process results in formation of the tooth crown shape, then the bud (E13.5), cap (E14.5), and bell (E16.5) stages. During tooth development, Sox2-positive (Sox2+) dental epithelial stem cells contribute to renewal of enamel-producing ameloblasts as well as all other epithelial cell lineages of the tooth germ^4^. For dental epithelial cell differentiation, signaling networks function with growth factors, transcription factors, adhesive molecules, and extracellular matrices to mediate this process^5^,^6^,^7^,^8^.

The basement membrane (BM), a sheet-like extracellular matrix, lies between epithelium and mesenchyme, and plays important roles in organogenesis by regulating signals for cell proliferation, migration, and differentiation^9^,^10^. BM cells contain type IV collagen, laminin, perlecan, and other molecules, though their structural composition in various tissues differs during the developmental stages. The biological activities of the BM can be largely attributed to laminins, which are major glycoprotein components. During tooth development, LAMA5 and LAMA2 play critical roles in tooth morphogenesis and cell differentiation^11^,^12^, while mutation of LAMA3 or LAMB3 can cause amelogenesis imperfecta^13^,^14^, suggesting that the BM has a role in regulation of tooth development and cell differentiation.

Nephronectin (Npnt), an ECM protein possessing 5 EGF-like repeat domains, as well as an RGD sequence and COOH-terminal MAM domain, localizes in the BM of developing organs, such as the eyes, lungs, teeth, hair, taste buds, and kidneys^15^. Lack of functional Npnt frequently results in kidney agenesis or hypoplasia, which can be traced to a delay in invasion of the metanephric mesenchyme by the ureteric bud during an early stage of kidney development^16^. As a BM molecule, Npnt is also required for the hair follicle stem cell niche that regulates arrector pili muscle cells^17^. Furthermore, it has been reported that Npnt is involved in BM assembly in association with the QBRICK protein^18^. Dysfunction of QBRICK provokes Fraser syndrome, which results in renal dysmorphogenesis, cryptophthalmos, syndactyly, dystrophic epidermolysis bullosa, and dental hypoplasia^19^,^20^,^21^, indicating that Npnt may play important roles in tooth development. In the present study, we found that Npnt plays critical roles in dental epithelial stem cell differentiation via regulation of Sox2 expression via the EGF signaling pathway through its EGF-like repeat domains.

## Results

### Npnt is highly expressed in tooth germ, and localized in the BM between dental epithelium and mesenchyme

**Figure 1:**
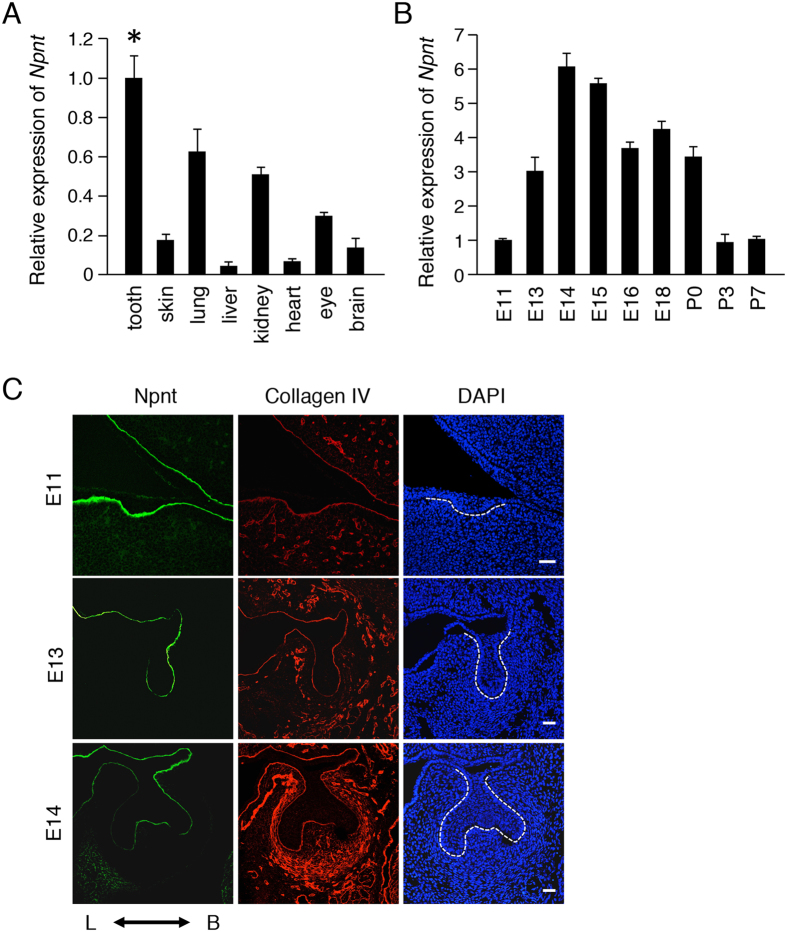
Npnt found to be highly expressed in developing teeth and localized in buccal BM of tooth germs. (**A**) qRT-PCR analysis of *Npnt* expression in teeth, skin, lungs, livers, kidney, heart, eyes, and brains of E14.5 embryos (n = 3). *Gapdh* was used as the internal control. *P < 0.05. Error bars represent mean ± S.D. (**B**) qRT-PCR analysis of *Npnt* expression in teeth obtained from mice on E11, E13, E14, E15, E16, E18, P0, P3, and P7. *Gapdh* was used as the internal control. (**C**) Npnt (green) and collagen IV (red) expressions in E11, E13, and E14 teeth, as detected by immunohistochemistry. Nuclei were stained with DAPI (blue). Scale bars: 50 μm. L; lingual side, B; buccal side.

In our previous study, genes specifically expressed in the tooth germ were identified using a differential display method^22^. In the present experiments, we found that Npnt is a BM molecule. To elucidate the expression pattern of Npnt during tooth development, we performed quantitative RT-PCR assays using total RNA from tooth, skin, lung, liver, kidney heart eye, and brain samples obtained on E14 and from teeth at various developmental stages [E11, E13, E14, E15, E16, E18, postnatal day 0 (P0), P3, P7]. The expression of Npnt was elevated in the tooth, lung, and kidney samples (Fig. 1A) as compared with those of other tissues, while its expression level was increased during the tooth morphogenesis stage (E13–E15) (Fig. 1B). These results indicate that Npnt plays important roles during tooth development, especially in morphogenesis.

To examine the localization of Npnt during tooth development, we performed immunohistochemistry examinations using lower first molars obtained on E11, E13, and E14, which indicated that it is localized in the BM during those developmental stages (Fig. 1C). On E13 and E14, Npnt was strongly expressed on the buccal side of the tooth germ, whereas the BM component collagen IV was uniformly expressed throughout the BM. These results suggest that Npnt is strongly expressed in teeth and localized in the BM on the buccal side of the developing tooth germ.

### Npnt expression pattern opposite of that of Sox2

**Figure 2:**
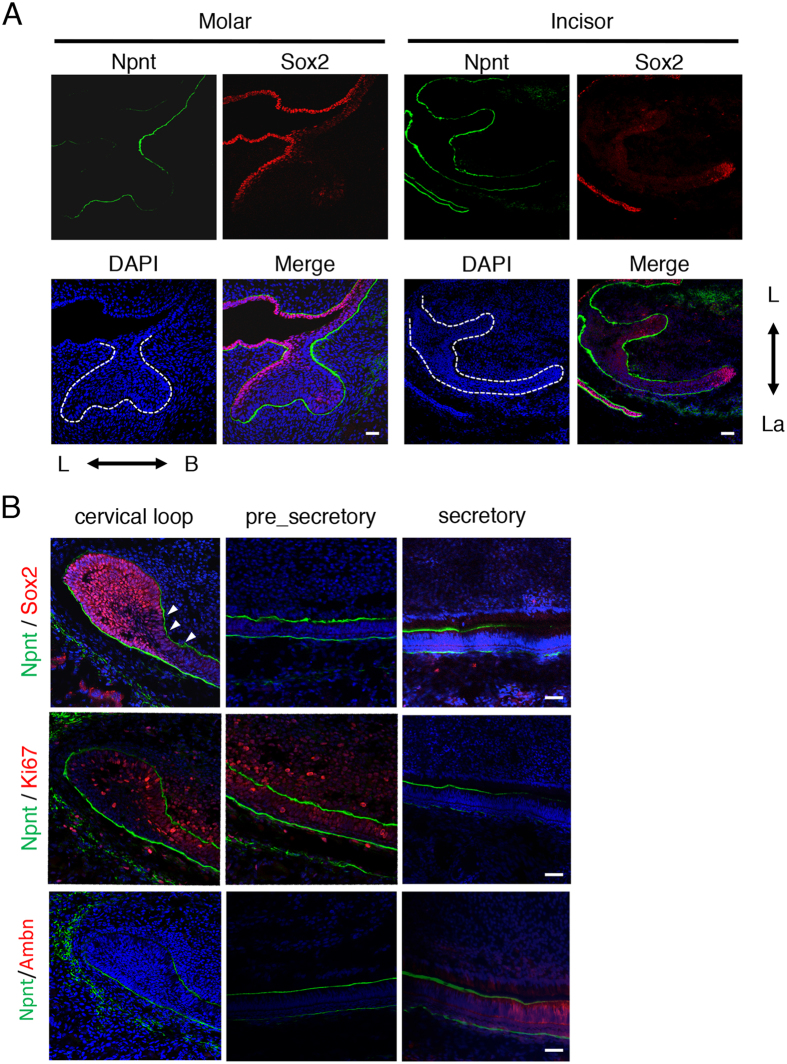
Npnt and Sox2 showed opposite expression patterns in molar and incisor cervical loop. (**A**) Expressions of Npnt (green) and Sox2 (red) in representative E14 molar and incisor, as detected by immunohistochemistry. Nuclei were stained with DAPI (blue). (**B**) Expressions of Npnt (green) and Sox2 (red) (upper panels), Npnt (green) and Ki67 (red) (middle panels), and Npnt (green) and Ambn (red) (lower panels) in representative P1 incisors, as detected by immunohistochemistry. Nuclei were stained with DAPI (blue). Arrowheads show area of Npnt expression, which was decreased in Sox2+ dental epithelial cells in the adjacent cervical loop. Scale bars: 50 μm. L; lingual side, B; buccal side, La; labial side.

Sox2+ dental epithelial cells are stem cells that have an ability to differentiate to all types of cells with a dental epithelial lineage, such as those of the inner dental epithelium, stratum intermedium, and outer enamel epithelium^4^. To examine Sox2 expression during tooth development, we performed immunohistochemistry experiments of developing teeth using antibodies for Sox2 and Npnt. Sox2+ cells were localized on the lingual side of E14 molars (Fig. 2A), whereas Npnt was expressed in the BM on the buccal side, suggesting that Npnt has an expression pattern opposite that of Sox2. We also used immunohistochemistry to examine E14 incisors. Mouse incisors continuously grow, due to the existence of a stem cell niche and cervical loop, which supply dental epithelial stem cells. Sox2+ cells were mainly localized on the apical end of the cervical loop (Fig. 2A). During dental epithelial cell differentiation, Sox2+ cells were decreased, while Npnt was increased in the BM between the IEE and dental mesenchyme in P1 incisors (Fig. 2B, arrowhead). To assess cell proliferation and differentiation, we stained with Ki67, a marker of cell proliferation, and the ameloblast differentiation marker ameloblastin (Ambn). Ki67-positive cells were increased in Sox2 decreased areas in the cervical loop of P1 incisors (Fig. 2B), which are termed transit-amplifying (T-A) cells. Also, Ambn was increased, while Npnt was decreased in the secretory stage of ameloblasts. Together, these results strongly suggest that Npnt regulates stem cell differentiation during dental epithelial development.

### Reduction of Npnt results in appearance of ectopic Sox2+ cells on buccal side of tooth germ in organ culture system

**Figure 3:**
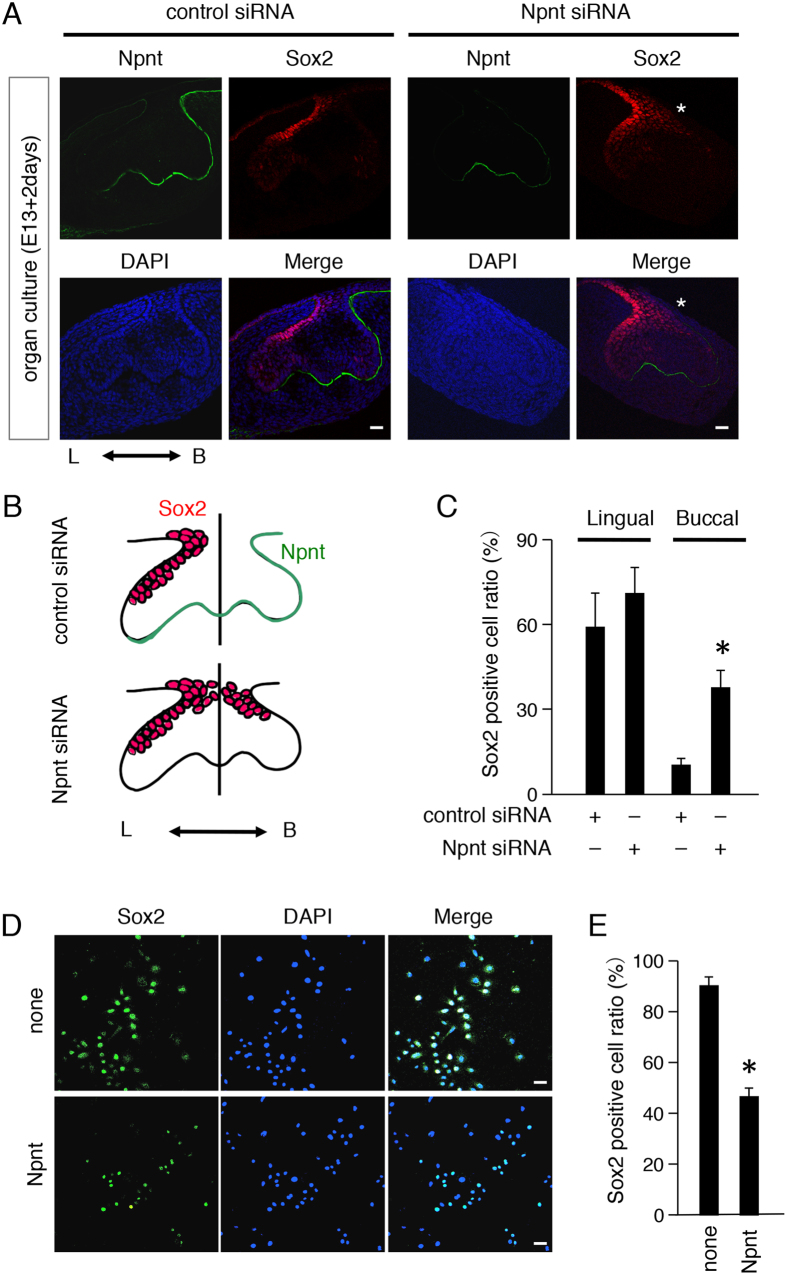
Npnt knockdown induced ectopic Sox2+ cells on buccal side of tooth germs. (**A**) Two-day organ cultures of representative E13 tooth germs transfected with control or Npnt siRNA. Npnt (green) and Sox2 (red) expressions were detected by immunohistochemistry. Nuclei were stained with DAPI (blue). (**B**) Schematic representation of cultured tooth germs. (**C**) Ratio of Sox2+ cells in cultured tooth germs divided into lingual and buccal sides following transfection with control siRNA or Npnt siRNA. (**D**) Sox2 (green) expression in M3H1 cells cultured in dishes with or without recombinant Npnt coating. Nuclei were stained with DAPI (blue). (**E**) Ratio of Sox2+ cells among M3H1 cells cultured with or without Npnt coating. The ratio was calculated as Sox2+ cells/DAPI-stained nuclei. *P < 0.05. Error bars represent mean ± S.D. Scale bars: 50 μm.

To examine the role of Npnt in Sox2+ cell distribution in developing teeth, we cultured E13 tooth germs with or without Npnt siRNA. Following 2 days of culture, tooth buds had grown to the cap stage and Npnt appeared on the buccal side of cultured tooth germs in the control siRNA group, while Sox2+ cells were predominant on the lingual side (Fig. 3A,B). Addition of Npnt siRNA decreased Npnt expression on the buccal side of the BM, while ectopic Sox2+ cells appeared on the buccal side of developing teeth. Furthermore, the ratio of Sox2+ cells was increased on the buccal side in the Npnt siRNA group (Fig. 3C). These results suggest that Npnt is critical for distribution of cells positive for Sox2 in tooth organogenesis. To elucidate whether Npnt regulates Sox2+ cells, we performed an Npnt coating assay using the dental epithelial stem cell line M3H1 cells. Those cells were generated from the cervical loop of lower incisors in mice at the age of 4 months old. We coated 12-well culture dishes with or without Npnt, then cultured M3H1 cells for 48 hours. Sox2+ cells were significantly reduced in the Npnt-coated dishes (Fig. 3D,E). These results suggest that exogenous Npnt functions as an extracellular matrix protein to regulate dental epithelial stem cell differentiation *in vitro*.

### Npnt regulates tooth germ size and cell proliferation

**Figure 4:**
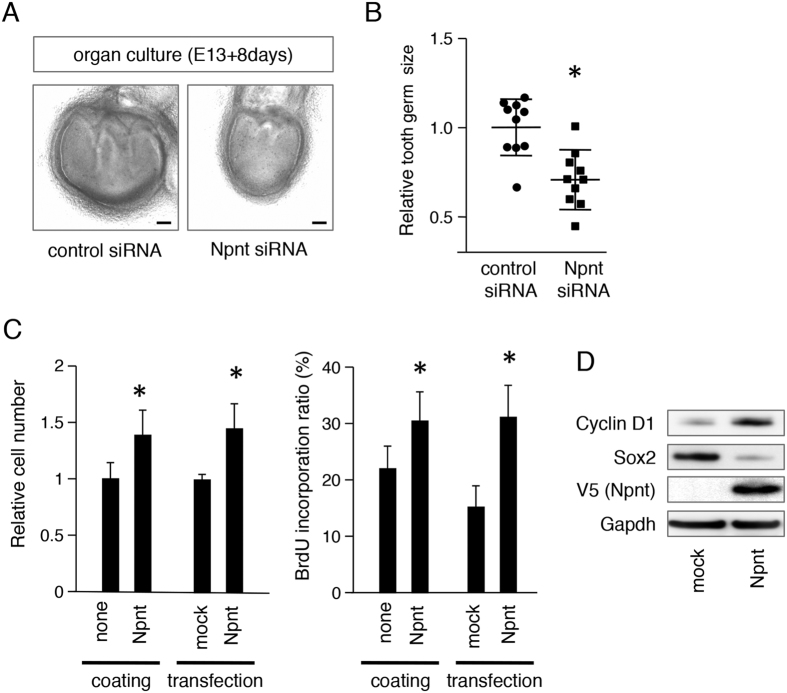
Npnt induced cell proliferation in *ex vivo* organ cultures and *in vitro*. (**A**) E13 tooth germs were transfected with control or Npnt siRNA and cultured for 8 days. (**B**) Relative tooth size plot (n = 10), with average tooth germ size in control siRNA group set at 1.0. (**C**) Cell proliferation was analyzed using a CCK-8 assay after coating the dishes with recombinant Npnt or transfection with Npnt. BrdU incorporation of M3H1 cells after coating dishes with recombinant Npnt or transfection with Npnt. The ratio was calculated as BrdU-positive cells/DAPI-stained nuclei. (**D**) Western blotting results of cyclin D1, Sox2, V5, and Gapdh in M3H1 cells transfected with mock vector or Npnt expression vector. Gapdh was used as the internal control. The full-length blots are presented in Supplementary Figure 1A. *P < 0.05. Error bars represent mean ± S.D. Scale bars: 100 μm.

To assess whether Npnt has effects on tooth germ development, we prepared an *ex vivo* organ culture system using Npnt siRNA. Tooth germs were dissected from E13 mice, then cultured for 8 days with or without Npnt siRNA. Tooth germ size was significantly reduced following Npnt knockdown as compared to control siRNA treatment (Fig. 4A,B). Next, we evaluated the proliferation of M3H1 cells using Npnt coating and Npnt transfection. Relative cell numbers were increased after 48 hours by both Npnt coating and Npnt transfection in comparison with the control cultures (Fig. 4C). BrdU incorporation was also increased by both Npnt coating and transfection. These results suggest that Npnt is necessary for epithelial cell proliferation. To examine the role of Npnt in cell cycle progression, we examined expression of the cell cycle molecule cyclin D1 under the condition of Npnt transfection using western blot analysis. Cyclin D1 was increased in M3H1 cells by Npnt overexpression (Fig. 4D), whereas Sox2 was reduced. These findings suggest that dental epithelial stem cell differentiation *in vitro* was induced by Npnt and the cell cycle was accelerated.

### EGF-like repeat domains of Npnt regulate cell proliferation and Sox2+ cell differentiation

**Figure 5:**
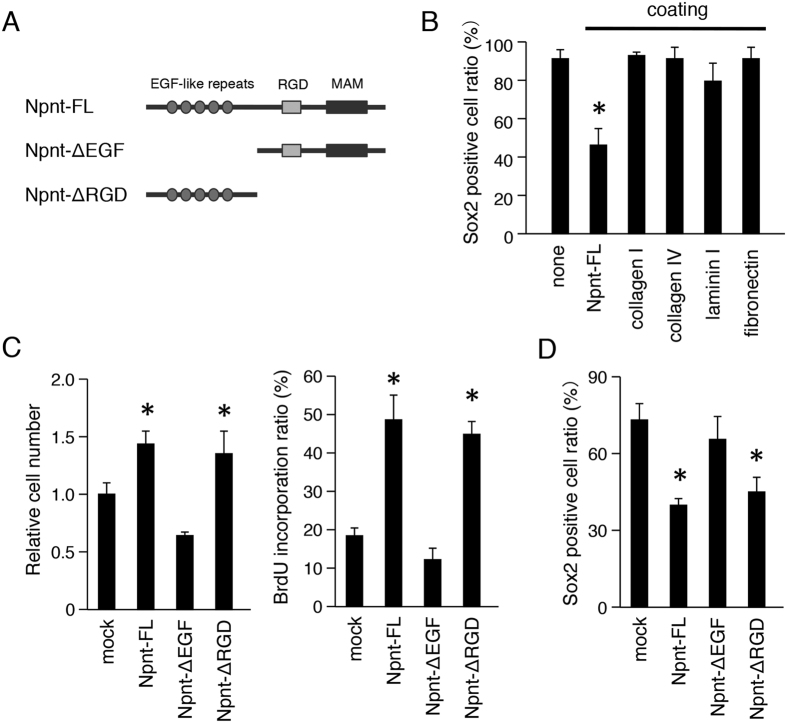
EGF-like repeats of Npnt are required for cell proliferation and Sox2 regulation. (**A**) Schematic representation of Npnt constructs. Npnt-FL, full-length Npnt; Npnt-ΔEGF, Npnt with deletion of EGF-like repeats domain; Npnt-ΔRGD, Npnt with deletion of RGD and MAM domains. (**B**) Ratio of Sox2+ cells among M3H1 cells in dishes coated with Npnt, collagen I, collagen IV, laminin I, and fibronectin. The ratio was calculated as Sox2+ cells/DAPI-stained nuclei. (**C**) Cell proliferation was analyzed using a CCK-8 assay after transfection with Npnt-FL, Npnt-ΔEGF, or Npnt-ΔRGD. BrdU incorporation analysis after transfection with Npnt-FL, Npnt-ΔEGF, or Npnt-ΔRGD. The ratio was calculated as BrdU-positive cells/DAPI-stained nuclei. (**D**) Ratio of Sox2+ positive cells among M3H1 cells transfected with Npnt-FL, Npnt-ΔEGF, or Npnt-ΔRGD. The ratio was calculated as Sox2+ cells/DAPI-stained nuclei. *P < 0.05. Error bars represent mean ± S.D.

To assess whether Sox2 regulation by Npnt is specific, we also tested other BM proteins including collagen I, collagen IV, laminin I, and fibronectin. Npnt coating reduced the number of Sox2+ cells, while coating with the other BM proteins did not have effects on Sox2 regulation (Fig. 5B). Npnt contains 5 EGF-like repeat domains, as well as an RGD sequence and MAM domain. To identify which domain is critical for cell proliferation and Sox2 regulation, we generated various constructs, including full length Npnt (Npnt-FL), Npnt lacking EGF-like repeats (Npnt-ΔEGF), and Npnt lacking the RGD and MAM domains (Npnt-ΔRGD) (Fig. 5A). Each of the expression vectors were transfected into M3H1 cells and culturing was performed for 48 hours. The cell number of the Npnt-FL transfectant was significantly increased in comparison with that of the mock transfectant (Fig. 5C), consistent the findings in our previous experiment (Fig. 4C). Furthermore, the cell number of Npnt-ΔEGF transfectant was significantly reduced in comparison with that of the Npnt-FL transfectant, whereas Npnt-ΔRGD showed a similar number of cells as compared with Npnt-FL (Fig. 5C). We also performed a BrdU incorporation assay to evaluate cell proliferation in each domain of the Npnt transfectants. Following transfection of Npnt-ΔEGF into M3H1 cells, the BrdU incorporation ratio was decreased in comparison with that of the Npnt-FL and Npnt ΔRGD transfectants (Fig. 5C). These findings suggest that the EGF-like repeat domains of Npnt, but not the RGD and MAM domains, are critical for cell proliferation in dental epithelial stem cells. Next, we examined whether Sox2+ cells are regulated by the EGF-like repeat domains of Npnt. The number of Sox2+ cells was significantly increased in Npnt-ΔEGF transfected cells in comparison with the Npnt-FL and Npnt-ΔRGD transfectants after culturing for 48 hours (Fig. 5D), revealing that the EGF-like repeat domains of Npnt regulate Sox2+ dental epithelial cell differentiation.

### EGF signaling is critical for Sox2 regulation by Npnt

**Figure 6:**
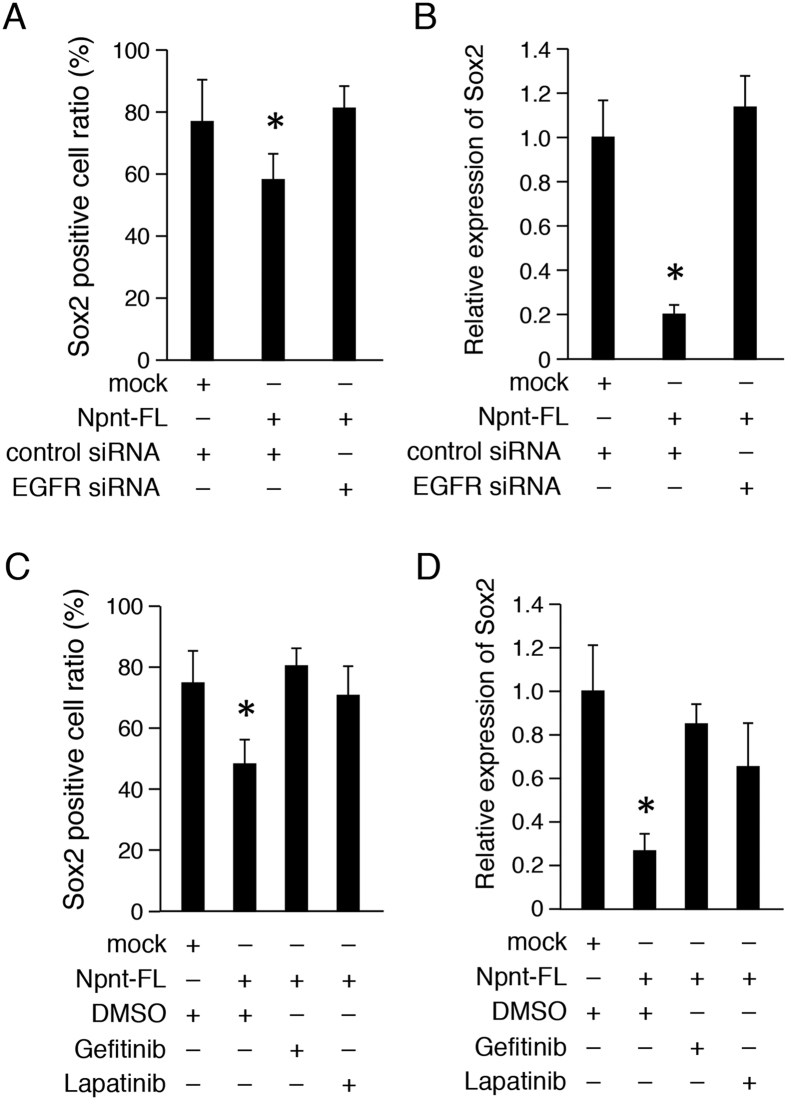
Npnt regulates Sox2 expression via EGF signaling pathway. (**A**) Ratio of Sox2+ cells among M3H1 cells transfected with Npnt-FL, with or without EGFR siRNA. The ratio was calculated as Sox2+ cells/DAPI-stained nuclei. (**B**) qRT-PCR analysis of *Sox2* expression in M3H1 cells transfected with Npnt-FL, with or without EGFR siRNA. *Gapdh* was used as the internal control. (**C**) Ratio of Sox2 cells among M3H1 cells transfected with Npnt-FL, or treated with EGFR inhibitor gefitinib or lapatinib. The ratio was calculated as Sox2+ cells/DAPI-stained nuclei. (**D**) qRT-PCR analysis of *Sox2* expression in M3H1 cells transfected with Npnt-FL, or treated with EGFR inhibitor gefitinib or lapatinib. *Gapdh* was used as the internal control. *P < 0.05. Error bars represent mean ± S.D.

It is known that EGF-like repeat domains play important roles in cell proliferation, differentiation, apoptosis, and morphogenesis by regulating the EGF signaling pathway^23^. To confirm whether the EGF-like signaling of Npnt is involved in Sox2 expression, we inhibited the EGF receptor (EGFR) by siRNA transfection. Sox2+ cells were reduced by overexpressed Npnt-FL, whereas that reduction was abrogated by transfection of EGFR siRNA into M3H1 cells (Fig. 6A). We also examined the mRNA expression of Sox2 using quantitative (q)RT-PCR, which revealed that its expression was decreased by Npnt-FL overexpression, while EGFR siRNA increased the level of Sox2 mRNA in M3H1 cells (Fig. 6B). Furthermore, we used the EGFR inhibitors gefitinib and lapatinib to assess the EGF signaling pathway. qRT-PCR findings of M3H1 cells showed that the ratio of Sox2+ cells was increased following stimulation by the EGFR inhibitors in comparison with stimulation by the Npnt-FL transfectant (Fig. 6C) and the mRNA expression level of Sox2 showed the same tendency (Fig. 6D). These results indicate that Npnt regulates Sox2+ cell differentiation via the EGF signaling pathway.

### EGF-like repeat domains of Npnt regulate EGF signaling pathway by regulating AKT phosphorylation in dental epithelial cells

**Figure 7:**
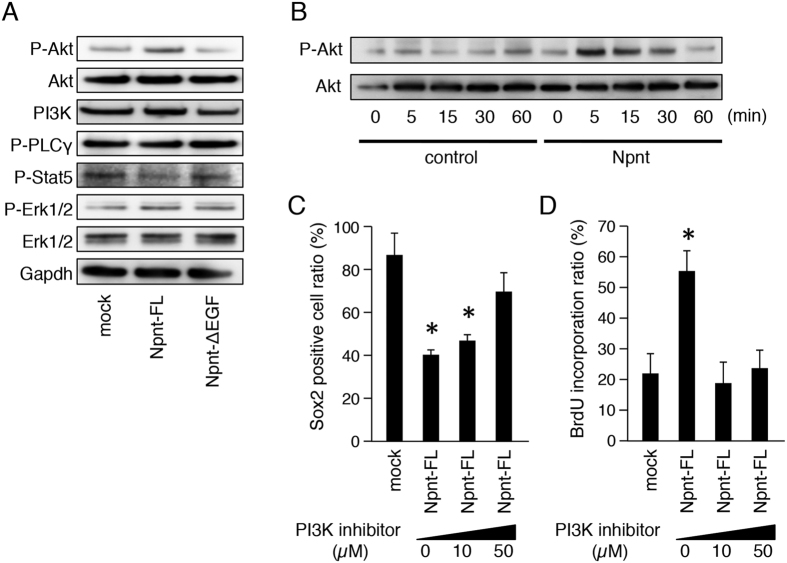
EGF-like repeats of Npnt regulate EGF signaling pathway by regulating phosphorylation of Akt. (**A**) Western blotting of P-Akt, Akt, PI3K, P-PLCγ, P-Stat5, P-Erk1/2, Erk1/2, and Gapdh in M3H1 cells transfected with Npnt-FL or Npnt-ΔEGF, and cultured for 48 hours. Gapdh was used as the internal control. The full-length blots are presented in Supplementary Figure 1B. (**B**) Western blotting of P-Akt and Akt in M3H1 cells with or without recombinant Npnt treatment for 60 minutes. Gapdh was used as the internal control. The full-length blots are presented in Supplementary Figure 1C. (**C**) Ratio of Sox2+ cells among M3H1 cells transfected with Npnt-FL and treated with different doses of PI3K inhibitor (LY294002). The ratio was calculated as Sox2 positive cells/DAPI-stained nuclei. (**D**) BrdU incorporation in M3H1 cells treated with different doses of LY294002. The ratio was calculated as BrdU-positive cells/DAPI-stained nuclei. *P < 0.05. Error bars represent mean ± S.D.

EGFR activates several signal transduction cascades, including the Akt, MAPK, and JNK pathways, which are involved in cell proliferation, differentiation, and cell survival. To identify which signaling pathway is activated by Npnt, we examined the phosphorylation of signal cascade molecules. M3H1 cells were transfected with the control or Npnt-FL transfectant, and an Npnt-ΔEGF expression vector, then phosphorylation of signaling molecules were evaluated by western blotting. After 48 hours of culture, Akt phosphorylation was increased in the Npnt-FL transfectant, but not in the Npnt-ΔEGF transfectant (Fig. 7A). On the other hand, P-Stat5 and P-Erk1/2 were not affected by either Npnt-FL or Npnt-ΔEGF transfection, suggesting that Akt signaling occurs downstream of Npnt activation in dental epithelial cells. For further analysis, we examined the short time activation of Akt by adding recombinant Npnt to the culture medium. As compared to the control, Akt phosphorylation was increased from 5 to 30 minutes after addition of recombinant Npnt (Fig. 7B). For confirmation, we inhibited PI3K, which is known to be upstream of Akt in the EGF signaling pathway, using a PI3K inhibitor (LY294002). Our findings showed that the ratio of Sox2+ cells in the Npnt-FL transfectant was increased by the addition of LY294002 in a dose-dependent manner (Fig. 7C), indicating that PI3K inhibition abrogates the reduction of Sox2+ cells caused by Npnt-FL overexpression. We also found that the increase in BrdU incorporation in the Npnt-FL transfectant was significantly reduced by adding LY294002 (Fig. 7D). Together, these results suggest that Npnt modulates Sox2+ cell differentiation in dental epithelial cells via the EGFR-PI3K-Akt signaling pathway.

## Discussion

The present results are the first to show that Npnt is required for Sox2+ dental epithelial stem cell differentiation and proliferation, including promotion of the EGF signaling pathway. Npnt was originally identified as an extracellular matrix molecule. POEM (pre-osteoblast epidermal growth factor-like repeat protein with meprin, A5 protein, and receptor protein-tyrosine phosphatase mu domain), which is associated with integrin a8b1^24^, is known to promote kidney development via integrin a8b1 mediated by the RGD domain^16^. Npnt also promotes osteoblast differentiation via EGF repeats^25^ and binds to heparin sulfate proteoglycan via its MAM domain^26^, while it also is involved in multipotent mechanisms as an extracellular scaffold via each of its domains. In the present study, we found that the EGF repeat domains of Npnt are critical for Sox2+ epithelial stem cell during tooth development.

EGF signaling is critical for self-renewal of human prostate cancer stem cells by regulation of Sox2 expression^27^. Ablation of EGFR, and of Src and Akt signaling suppresses self-renewal growth and expansion of stem-like side-population cells in non-small cell lung cancer^28^, indicating that EGF signaling is required for cancer stem cell maintenance. Postnatal brain specimens from mice lacking either Sox2 or Egfr were found to exhibit similar defects^29^,^30^, indicating that both Sox2 and EGF signaling are required for maintenance and expansion of neural precursor cells. Our experiments demonstrated that the EGF-like repeats of Npnt can activate the EGF signaling pathway to regulate Sox2 expression in dental epithelial cells. It was previously reported that EGF-like repeats induce EGFR phosphorylation and activate EGF signaling as EGFR ligands^31^, indicating that Npnt directly activates the EGF signaling pathway via binding with EGFR during tooth development.

EGF is well known to activate multiple signaling branches, including the PI3K/Akt, ERK, and JAK/STAT pathways^23^,^32^. Our findings indicate that the PI3K/Akt pathway, which is involved in cell growth apoptosis and migration^33^,^34^, plays roles in dental epithelial stem cell differentiation regulated by Npnt. PI3K, a dimeric enzyme composed of a regulatory p85 subunit, is responsible for anchorage to erbB receptor-specific docking sites and a catalytic p110 subunit that generates the second messenger phosphatidylinositol 3, 4, 5-serine/threonine kinase Akt^33^. It has also been reported that Sox2 causes an increase in activated Akt and activated Akt signaling is part of the negative feedback loop that assists Sox2 suppression in embryonic stem cells^35^, indicating that Sox2 transcription is carefully controlled by Akt signaling.

Sox2 is a transcription factor required for maintenance of pluripotency, while it also plays an essential role in different types of multipotent stem cells^36^,^37^,^38^ and induction of pluripotency^39^. A previous study analyzed dental epithelial cells from the cervical loop in mice during tooth development, and found them to contain a stem cell niche for all lineages of dental epithelial cells, with Sox2 shown to be a specific marker for those stem cells^4^. In another study of tooth development, it was reported that deletion of Smad4 in dental epithelium resulted in ectopic activation of SHH-Gli1 signaling and ectopic Sox2+ epithelial stem cells, indicating that the BMP-SHH signaling network regulates Sox2+ epithelial stem cells in teeth^40^. Previously, we reported that Ca^2+^ dramatically decreased Sox2+ dental epithelial stem cells *in vitro*, and also showed that ablation of mediator subunit 1 (MED1) resulted in switching of Sox2+ dental epithelial stem cell fate and contributed to ectopic hair formation in teeth^41^. These results indicated that Sox2+ cells are governed by differential signaling, which can control their cell fate. In the present study, Npnt reduced the number of Sox2+ stem cells and increased cell proliferation by regulating the EGF signaling pathway, which strongly suggests that Npnt contributes to commitment of Sox2+ dental epithelial stem cell differentiation to an ameloblast cell lineage and promotes cell proliferation, indicating that Npnt is useful for ameloblast cell maintenance and culture systems as an extracellular matrix protein.

This is the first report to describe the roles of Npnt during tooth development. Our findings reveal the mechanisms of Npnt, and suggest possible applications for controlling dental epithelial stem cell differentiation and proliferation by regulating extracellular molecules.

## Experimental Procedures

### Tissue preparation and histological analysis

Pregnant mice were euthanized by anesthesia and their embryos immediately dissected. Embryo heads were fixed with 4% paraformaldehyde in phosphate-buffered saline (PBS) for 16 hours at 4 °C, then embedded in O.C.T. compound (Sakura Finetek, Tokyo, Japan). Frozen sections of mandibular molars and incisors were obtained from mouse heads at developmental stages (E11, E13, E14, P1). All animal experiments were approved by the ethics committee of Kyushu University Animal Experiment Center (protocol no. A21–103–0). All procedures were performed in accordance with the relevant guidelines and regulations. Immunohistochemistry was performed using primary antibodies to Npnt (1:500, R&D System), Sox2 (1:250, abcam), Ambn (1:500, Santa Cruz), Ki67 (1:250, Cell Signaling), and Collagen IV (1:500, Millipore) for 16 hours at 4 °C. The sections were then incubated with secondary antibodies conjugated with Alexa 488 or Alexa 594 fluorescent dye for 1 hour at room temperature. To visualize nuclei, sections were mounted with Vectashield mounting medium containing DAPI (1:1000, VECTASHIELD mounting medium hard set with DAPI). Images were captured with a C2 confocal microscope (Nikon, Tokyo, Japan) and analyzed using NIS-Elements AR software v4.00 (Nikon).

### RNA isolation and qRT-PCR analysis

Total RNA was isolated from E14 mouse tissues (tooth, skin, lung, liver, kidney, heart, eye, brain) and molar tooth buds during developmental stages (E11, E13, E14, E15, E18, P0, P3, P7) using TRIZOL reagent (Life Technologies), then purified using a RNeasy Mini kit (QIAGEN). cDNA was synthesized using SuperScript III reverse transcriptase reagent (Life Technologies). The specific forward and reverse primers used for qRT-PCR were as follows: *Npnt*, 5′-aagtgccctatcgtgttcca-3′ and 5′-gcagatgtagctcccaaacg-3′; *Sox2*, 5′-ggcaatcaaatgtccatt-3′ and 5′-tccttccttgtctgtaac-3′; and *glyceraldehyde 3-phosphate dehydrogenase (Gapdh*), 5′-ggagcgagaccccactaacatc-3′ and 5′-ctcgtggttcacacccatcac-3′. Expression of each gene was normalized to that of *Gapdh*. qRT-PCR was performed using iQ SYBR Green Supermix (Bio-Rad) with a CFX Connect Real-Time PCR detection system (Bio-Rad).

### Cell culture and transfection

M3H1 cells, a dental epithelial cell line, were established as we previously reported^41^. Briefly, dental epithelial cells were harvested from the cervical loop of mandible incisors obtained from 4-month old mice. Cells were cultured in Ca^2+^-free keratinocyte serum-free medium (K-SFM, 37010–022; Gibco/Life Technologies) supplemented with epidermal growth factor, bovine pituitary extract, and 1% penicillin/streptomycin (Gibco/Life Technologies) for 1 month without passage at 37 °C in a humidified incubator in an atmosphere containing 5% CO_2_, with the medium changed every 3 days. After 1 month, epithelial cell-like colonies was harvested and ameloblastic characterization was confirmed. For transfection, cells were cultured in 12-well plates at a density of 2 × 10^5^ cells/well in K-SFM, then transfected with the expression vector using Lipofectamine 3000 with Plus reagent (Life Technologies), according to the manufacturer’s protocol. For short interfering (si)RNA transfection, Lipofectamine 3000 without Plus reagent was applied. siRNAs for Npnt (ON-TARGET Plus L-049072-01-0005; Dharmacon) and Egfr (ON-TARGET Plus L-040411-00-0005; Dharmacon), as well as the control siRNA (ON-TARGET Plus Non-targeting Control Pool D-001810-10; Dharmacon) were purchased. To inhibit EGF signaling, gefitinib (1 μM, Sigma Aldrich) and lapatinib (1 μM, Sigma Aldrich) were applied to M3H1 cells. For a PI3K inhibiting assay, a PI3K inhibitor (LY294002, Cell Signaling Technology) was used at concentrations ranging from 0-50 μM.

### Organ culture

Mandibular molar tooth germs were dissected from E13 mice embryos, seeded into a cell culture insert (BD Falcon; BD Biosciences), and grown using an air-liquid interface culture technique in Dulbecco’s Modified Eagle’s Medium/F12 supplemented with 20% fetal bovine serum (Gibco/Life Technologies), 180 μg/ml ascorbic acid, 2 mM l-glutamine, and 50 U/ml penicillin/streptomycin at 37 °C in a humidified atmosphere of 5% CO_2_ for 8 days, as previously described^7^,^42^. For siRNA-mediated knockdown, cells were transfected with control or Npnt siRNA at a concentration of 500 nM using Lipofectamine 3000, according to the manufacturer’s protocol. The knockdown efficiency of Npnt siRNA for tooth germs was confirmed by qRT-PCR and western blotting assay (Supplementary Fig. 2A, B). Cultured organs were embedded in frozen blocks, then sections were immunostained with Npnt and Sox2 antibodies. Tooth germs were divided into the lingual and buccal sides (see Fig. 3B), and the ratio of Sox2+ cells was determined under a fluorescence microscope. For evaluation of tooth size, E13 tooth germs were cultured for 8 days, then size was determined using Image J software.

### Recombinant protein coating analysis

We coated 96-well plates overnight at 4 °C with recombinant Npnt (5 μg/ml, R&D Systems), collagen I (10 μg/ml, Nippi), collagen IV (10 μg/ml, Nippi), laminin I (10 μg/ml, Nippi), and fibronectin (10 μg/ml, Nippi), then they were washed with PBS and blocked with 3% bovine serum albumin for 1 hour at 37 °C. M3H1 cells were then plated at a concentration of 2 × 10^4^ cells/well and cultured for 48 hours. Sox2+ cells were determined by immunohistochemistry using an anti-Sox2 antibody. Total cells were counted using DAPI, then the ratio of Sox2+ cells was determined under a fluorescence microscope.

### Construction of expression vectors

Npnt expression vectors were constructed using a Gateway cloning system (Life Technologies), according to the manufacturer’s protocol. Briefly, the coding sequence of mouse Npnt without a stop codon was cloned into a pENTR/D-TOPO vector. The following forward and reverse primers were used: Npnt-FL, 5′-caccatggctgtgctcctagcggcggt-3′ and 5′-gcagcgacctcttttcaagctcac-3′; Npnt-ΔEGF, 5′-caccatggctgtgctcctagcggcggt-3′ and 5′-aggaatcctattcgcatgtcct-3′; and Npnt-ΔRGD, 5′-caccatggatgctggaagtacaaggt-3′ and 5′-gcagcgacctcttttcaagctcac-3′. The expression vectors were cloned using an LR recombination reaction (pcDNA-DEST40; Life Technologies), then tagged with V5-His.

### Cell proliferation and bromodeoxyuridine (BrdU) incorporation

M3H1 cells were transfected with an Npnt expression vector, then cultured in Npnt-coated dishes for 48 hours. Proliferation was determined using a Cell Counting Kit (CCK)-8 (Dojindo Laboratories), according to the manufacturer’s protocol, while BrdU incorporation was assayed using a BrdU labeling kit (Roche Diagnostics). After 48 hours of culture, BrdU was applied to the plates for 30 minutes, then incorporated BrdU was detected after washing with PBS, according to the manufacturer’s protocol. BrdU-positive cells were counted under a fluorescence microscope.

### Western blotting

For detection of phosphorylation of each signaling molecule, cells were washed twice with 1 mM ice-cold sodium orthovanadate (Sigma-Aldrich) in PBS, then lysed with CelLytic M (Sigma-Aldrich) supplemented with a 1% protease inhibitor cocktail (Sigma-Aldrich) and 1 mM phenylmethylsulfonyl fluoride (Sigma-Aldrich). Protein from each sample (10 μg) was separated using 4–12% sodium dodecyl sulfate-polyacrylamide gel (NuPAGE: Invitrogen) and analyzed by western blotting. Blotted membranes were incubated with antibodies for cyclin D1 (1:500, Cell Signaling Technology), Sox2 (1:500, Abcam), P-Akt (1:500, Cell Signaling Technology), Akt (1:500, Cell Signaling Technology), PI3K (1:500, Cell Signaling Technology), P-PLCγ (1:500, Cell Signaling Technology), P-Stat5 (1:500, Cell Signaling Technology), P-Erk 1/2 (1:500, Cell Signaling Technology), Erk 1/2 (1:500, Cell Signaling Technology), and Gapdh (1:500, Cell Signaling Technology), then signals were detected with an ECL kit (Amersham Biosciences) and visualized using an Image Quant LAS 4000 system (GE Healthcare, Life Science).

### Statistical analysis

All experiments were repeated at least 3 times to confirm reproducibility and the data was analyzed using Prism 6 software (GraphPad Software, La Jolla, CA, USA). Statistical significance was determined using a two-tailed unpaired Student’s t test for differences between two groups of data. One-way ANOVA was used for the quantification between multiple groups. Differences with P values < 0.05 were considered to be statistically significant.

## Additional Information

**How to cite this article:** Arai, C. *et al*. Nephronectin plays critical roles in Sox2 expression and proliferation in dental epithelial stem cells via EGF-like repeat domains. *Sci. Rep.* **7**, 45181; doi: 10.1038/srep45181 (2017).

**Publisher's note:** Springer Nature remains neutral with regard to jurisdictional claims in published maps and institutional affiliations.

## Supplementary Material

Supplementary Figures
