# Supplementary material for: Nephronectin plays critical roles in Sox2 expression and proliferation in dental epithelial stem cells via EGF-like repeat domains
Source: Sci Rep. 2017 Mar 27;7:45181. doi: 10.1038/srep45181 (PMC5366923; doi:10.1038/srep45181)
Supplement: Supplementary Figures [file srep45181-s1.pdf]

Nephronectin plays critical roles in Sox2 expression and proliferation in dental epithelial stem cells via EGF-like repeat domains

Chieko Arai[1 ‡], Keigo Yoshizaki[1 ‡], Kanako Miyazaki[1], Kan Saito[2], Aya Yamada[2], Han Xue[1], Keita Funada[1], Emiko Fukumoto[2], Naoto Haruyama[1], Tsutomu Iwamoto[3], Ichiro Takahashi[1], and Satoshi Fukumoto[2]*

Supplementary Figure 1

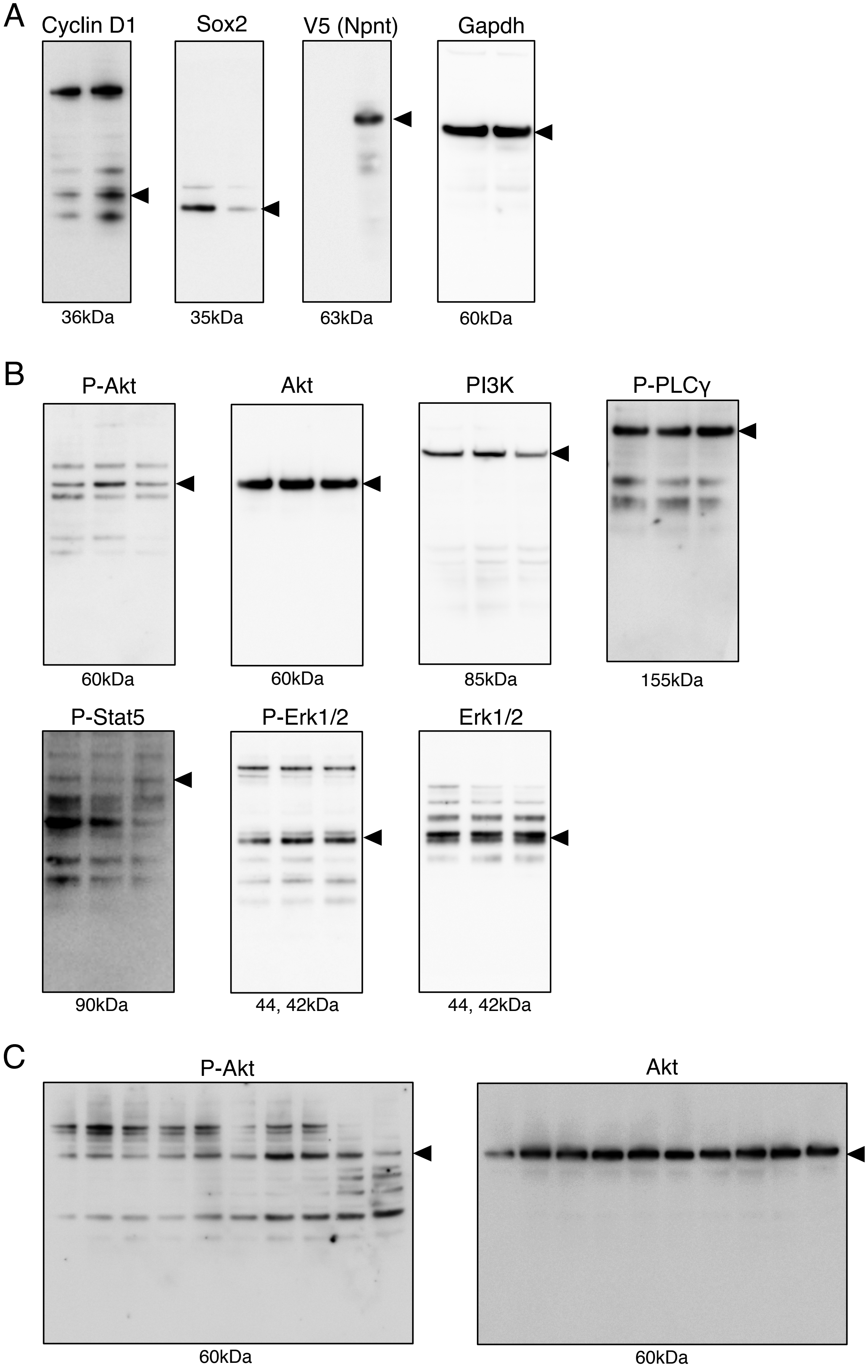

Supplementary Figure 2

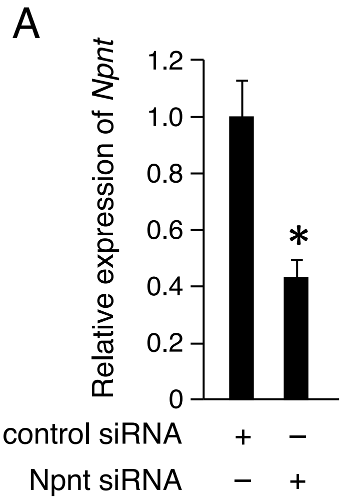

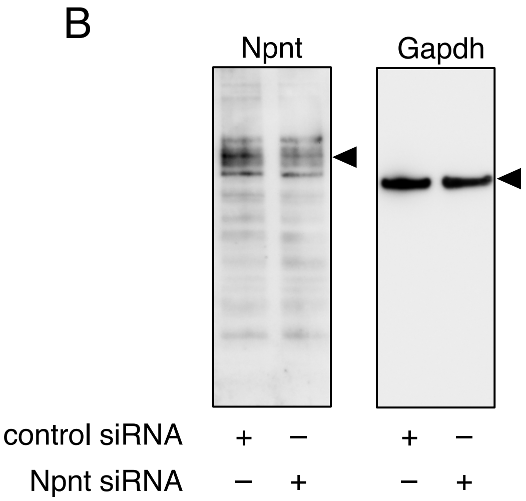

Supplemental Figure 1. Full-length pictures of western blotting. Full-length blots for Fig. 4D (A), Fig. 7A (B) and Fig. 7B (C). Arrow heads represent each band as expected size.

Supplemental Figure 2. The knockdown efficiency of Npnt siRNA for tooth organ culture. A. qRT-PCR analysis of Npnt expression in cultured tooth transfected with control siRNA or Npnt siRNA. *Gapdh* was used as the internal control. B. Western blotting of Npnt and Gapdh in cultured teeth transfected with control siRNA or Npnt siRNA, and cultured for 24 hours. Gapdh was used as the internal control.
